# Regulation of bacterial phosphorelay systems

**DOI:** 10.1039/d5cb00016e

**Published:** 2025-06-19

**Authors:** Daniel M. Foulkes, Daniel M. Cooper, Catherine Westland, Dominic P. Byrne

**Affiliations:** a Department of Biochemistry, Cell and Systems Biology, Institute of Systems, Molecular and Integrative Biology, University of Liverpool Liverpool L69 7ZB UK bs0u4193@liverpool.ac.uk

## Abstract

In terms of biomass, bacteria are the most successful organisms on earth. This is partly attributed to their tremendous adaptive capabilities, which allows them to sense and rapidly organise responses to changing environmental stimuli. Using complex signalling mechanisms, bacteria can relay cellular information to fine-tune their metabolism, maintain homeostasis, and trigger virulence processes during infection. Across all life, protein phosphorylation represents the most abundant signalling mechanism, which is controlled by a versatile class of enzymes called protein kinases and their cognate phosphatases. For many years, histidine kinase (HK)-containing two-component systems (TCSs) were considered the canonical instruments of bacterial sensing. However, advances in metagenomics has since proven that bacterial phosphorelay is in fact orchestrated by a functionally diverse array of integrated protein kinase types, including Ser, Thr, Tyr and Arg-targeting enzymes. In this review, we provide an up-to-date appraisal of bacterial kinase signalling, with an emphasis on how these sensing pathways are regulated to modulate kinase output. Finally, we explore how selective kinase inhibitors may be exploited to control infections and combat the looming health emergency of multidrug resistant bacteria.

## Abstract 2

This review examines the regulatory mechanisms governing the catalytic activities of diverse classes of bacterial kinases and evaluates their viability as targets for the development of novel antimicrobial strategies.

## Graphical abstract

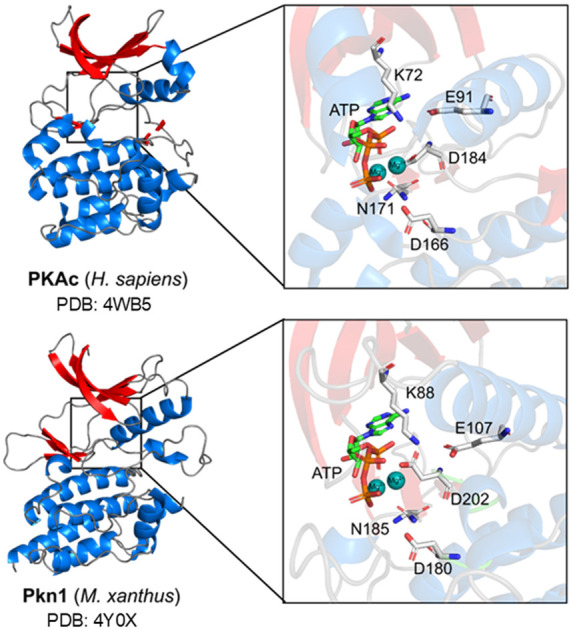

Life has evolved an abundance of protein-based molecular machines to detect and coordinate responses to environmental stimuli. These biological processes are collectively referred to as signal transduction events and are controlled, in part, by a series of functionally divergent enzymes that post-translationally introduce covalent chemical modifications onto target proteins in a tightly regulated manner. One such class of enzymes are the protein kinases, which catalyse the reversible addition of a phosphate group from ATP to conserved amino acid residues within a protein substrate, in a process called phosphorylation. Since the discovery of protein kinase activity in 1954,^1^ and the first protein kinase (phosphorylase kinase) a few years later,^2–4^ it is now accepted that protein phosphorylation controls virtually every aspect of life. Indeed, protein phosphorylation is the most extensively studied post-translational modification in bacteria, as evidenced by the wealth of literature written on the topic.^5–13^ Bacterial kinases can be classified into 5 groups: His kinases (HKs), non-Hanks type Tyr kinases, Arg kinases, Hanks-type Ser/Thr kinases (the so-called ‘eukaryotic-like Ser/Thr kinases’ [eSTKs]), and atypical Ser/Thr kinases.^14^ HK-containing two-component systems (TCS) constitute the dominant signal transduction circuitry across all prokaryote lineages and function as conduits between extracellular sensing and intracellular responses. Individual bacterial species can encode multiple TCSs, which enables sensing of a diverse and ever-expanding repertoire of known environmental signals.^15–17^ The ubiquitous TCSs are thus considered master regulators of prokaryotic metabolism,^18–20^ adaptation to environmental changes,^21–23^ virulence,^18,24–27^ antimicrobial resistance,^28–33^ motility and chemotaxis.^34–37^ Although TCSs are not components of mammalian cellular signalling networks, similar systems have been identified in some eukaryotic fungi, and higher plants.^38^ These eukaryotic TCSs are structurally more divergent, often supplemented with additional regulatory modules, and can tune the outputs of downstream signalling proteins, such as other kinases.^38–41^

In addition to HKs, phosphorylation of protein Ser/Thr and Tyr residues (*O*-phosphorylation), by enzymes that share striking sequence or structural similarities with canonical eukaryotic Hanks-type kinases (Fig. 1), has also emerged as a pervasive phosphosignalling mechanism in bacteria and archaea.^42,43^ Since the discovery of the first eSTK, Pkn1 in *Myxococcus xanthus*,^44^ it is now established that the genomes of bacteria can contain multiple eSTKs, with the prevalence of these kinases approximately correlating with the complexity of the bacteria's replicative niche.^45^ For example, *Sorangium cellulosum* encodes 317 eSTKs, which may imply a multi-directional signalling network reminiscent of eukaryotic systems.^46^ In fact, >80% of the *Mycobacterium tuberculosis* proteome (which encodes 11 eSTKs, including PknG (Fig. 1B)), and 63% of the theoretical proteome of *Clostridium difficile* is predicted to be *O*-phosphorylated.^7,12^ Several bacteria also encode atypical Ser/Thr protein kinases which diverge from the traditional Hanks-type kinase fold. For example, the HPr kinase/phosphorylase (HprK/P) of *Bacillus subtilis* is unrelated to Hanks-type kinases (instead binding nucleotides *via* a Walker A motif located in the P-loop) and regulates catabolite repression by phosphorylating (and dephosphorylating) the central HPr protein of the phosphoenolpyruvate: sugar phosphotransferase system.^47–49^ YihE is another example of an atypical Ser/Thr kinase of *E. coli* that does not resemble canonical eSTKs in terms of structure or sequence homology, but plays critical role in regulating stress-related programmed cell death in bacteria.^50,51^ Interestingly, CotH, an atypical protein kinase of *B. subtilis* and *B. cereus* (Fig. 1C) shares sequence similarity with the human Golgi casein kinase, Fam20C.^52^ Although CotH is not known to serve sensing or signalling functions *per se*, it is required to maintain the integrity of the spore-coat structure.^52^ CotH retains some features of canonical Ser/Thr protein kinases (including conserved active site residues corresponding to D166, N171, and D184 of the prototypical protein kinase, PKA) but diverges from the typical kinase fold in several crucial ways (Fig. 1C and D); it lacks a putative metal binding DFG motif and utilises an unusual mode of ATP binding involving two aromatic residues, Tyr142 and Trp245, that ‘sandwich’ the adenine moiety of ATP.^52^ In contrast to eSTKs, bacterial tyrosine kinases (BY tyrosine kinases) display limited homology with their mammalian equivalents, and are characterised by Walker A and B ATP/GTP binding motifs.^53^ Finally, while “classical” sensing and signalling roles have yet to be identified, arginine phosphorylation is known to regulate several cellular processes, particularly in Gram-positive bacteria, such as *B. subtillus* and *Staphylococcus aureus*, where it functions as a protein degradation signal.^54,55^ For example, phosphorylation of proteins by the arginine kinase, McsB of *B. subtilis*, targets them for recycling by the ClpCP protease system.^54^ In contrast to the other phosphotransfer systems, *N*-phosphorylation of arginine is a relatively understudied PTM, owing to the intrinsic lability of the phosphoamidate bond impeding conventional proteomics investigations.^56^ As a result, our broader understanding of arginine phosphorylation and its roles within bacterial signaling networks remains limited. Collectively, the varied phosphorelay systems encoded by bacterial species provide a finely tuned and tightly regulated series of signal cascades that govern multiple aspects of bacterial life. This review provides an up-to-date account of the mechanisms that regulate bacterial phosphotransfer events.

**Fig. 1:**
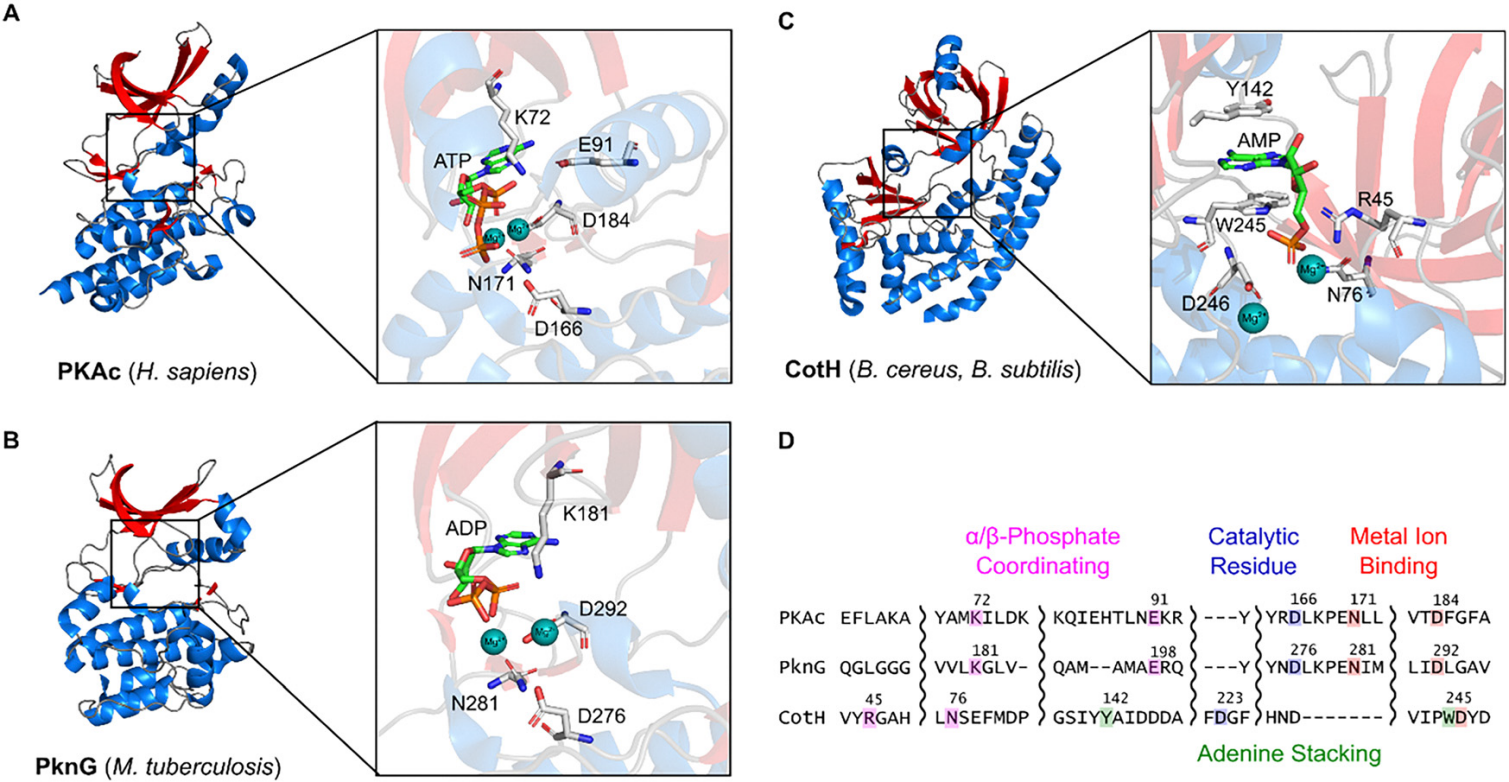
Structural similarities between eukaryotic and prokaryotic Ser/Thr kinases. Crystal structures of (A) human PKA in complex with ATP (PDB: 4WB5), (B) *M. tuberculosis* PknG in complex with ADP (4Y0X) and (C) *Bacillus* atypical protein kinase, CotH, in complex with AMP. Insets show ATP/AMP coordinating amino acid residues (white sticks). α-Helices are shaded blue, β-strands are shaded red, loops are shaded grey, Mg^2+^ ions are represented as turquoise spheres and ATP/AMP is represented by green sticks. (D) Sequence alignment of PKA with PknG and CotH (using MUSCLE). Canonical amino acid residues (or functional equivalents) involved in ATP binding or phosphate transfer are colour coordinated. Note that the atypical kinase, CotH, aligns poorly due to its highly diverged sequence.

## Regulation of TCS phosphorelay signals

**Fig. 2:**
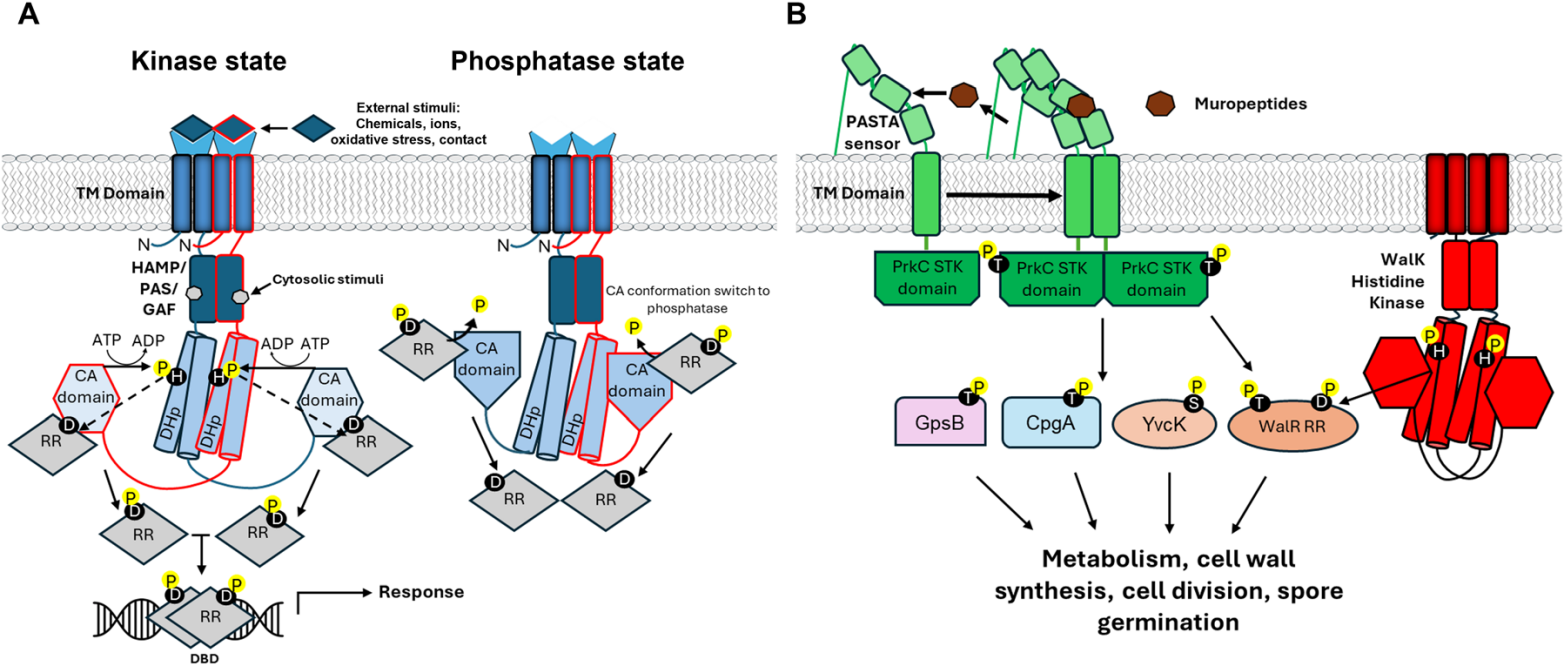
Overview of bacterial kinase signal transduction mechanisms. (A) Asymmetrical TCS kinase state: classical two component signalling systems (TCSs) respond to extracellular (through periplasmic receptor) or cytosolic (through HAMP/PAS/GAF) sensor domains, leading to dimerization. In a multistep phosphorelay, the catalytic CA domains phosphorylate, in *trans*, a conserved His in the DHp domain. The phosphate is then shuttled (dashed arrows) to a conserved Asp residue in cognate response regulators (RRs). RRs dimerize and bind to a specific DNA binding domain (DBD) to elicit transcription of target genes. Symmetrical TCS phosphatase state: in the absence of stimuli, the CA domain undergoes a conformation switch to a phosphatase-active state, dephosphorylating RRs in *cis*, releasing phosphate into the cytosol. (B) Simplified schematic of a *B. subtilis* eSTK, PrkC, responding to extracytosolic stimuli *via* PASTA sensor domains. During stationary phase, binding of muropeptides to PASTA repeats induces dimerization of PrkC TM and eSTK domains, leading to in *trans* autophosphorylation and subsequent phosphorylation of a range of target cytosolic proteins. Substrates include, but are not limited to, GpsB (a cell division protein^74^), YkwC (an oxidoreductase^75^) and CpgA (a GTPase^76^). PrkC also regulates the WalRK TCS by directly phosphorylating the WalR RR, influencing expression of genes relating to cell wall biosynthesis.^77^

A prototypical bacterial histidine kinase (HK) is a multidomain His to Asp phosphorelay enzyme (Fig. 2A), consisting of a variable N-terminal “sensing region”, transmembrane (TM) domain, and a conserved catalytic transmitter domain (containing catalytic ATPase [CA domain] and homodimeric phosphoacceptor/phosphotransferase [DHp] subdomains^57^). HKs also commonly contain one or more intracellular signalling domains; including HAMP (found in histidine kinases, adenylyl cyclases, methyl binding proteins, and phosphatases), PAS (Per-ARNT-Sim), STAC (SLC and TCST-Associated Component) or GAF (found in cGMP-specific phosphodiesterase, adenylyl cyclases, and FhlA) domains,^57,58^ that augment HK regulation by serving as sensing conductors of extracellular stimuli. Upon stimulation, the signal is transduced to the TCS catalytic core, resulting in ATP binding by the CA domain and autophosphorylation of the conserved DHp acceptor His residue. The phosphate moiety is then relayed to the receiver (REC) domain of a response regulator (RR) module, which catalyses its own aspartic acid phosphorylation.^38^ Following phosphorylation, most RRs undergo a conformational switch (typically leading to homodimerisation) which facilitates binding to regulatory genomic motifs and activation or repression of gene expression.^59–62^ Fascinatingly, there is also growing evidence to suggest that some RRs also possess phosphorylation-independent regulatory functions.^63–65^ For example, the phosphorylated form of the *Salmonella typhimurium* RR, RcsB, can homodimerize or form a heterodimer with the ancillary regulator, RcsA, to modulate gene transcription.^66^ However, recent structural investigations have revealed that an active RcsB dimer can be stabilized just by binding to DNA even in the absence of phosphorylation^67^ and the unphosphorylated form of RcsB can also interact with other transcription factors to regulate gene expression^68–71^ adding an extra layer of regulatory complexity to the traditional TCS model. Some HKs are also ‘hybrid kinases’ that are supplemented with carboxy-terminal REC and/or histidine phosphotransfer (HPt) domains, which are frequently involved in multi-step phosphorelay systems with contiguous signalling elements, such as DHp and RR proteins.^72^ Although TCS-HKs are canonically recognized to adopt a dimeric architecture, monomeric species have also been reported, including EL346 of *Erythrobacter litoralis*.^73^ In the inactive EL346 state, the typical DHp dimer interface of the kinase domain is blocked by direct interaction with a blue-light sensitive light–oxygen–voltage (LOV) domain, which competes with the CA domain for access to the phosphorylatable histidine.

Extra/intracellular or periplasmic signals are typically sensed by N-terminal located sensory PAS domains and propagated along the HK through a series of transient structural rearrangements and inter-domain interactions that regulate catalytic activity and phosphotransfer.^58,78^ Although the amino acid composition between different PAS domains of HKs is extremely variable (which drives substrate specificity), they adopt a highly conserved multi-β-sheet core structure with adjoining α-helices that forms a ligand-accepting cleft region.^57,78,79^ Sensing can also be achieved by PAS-like domains, all α-helix type structures, and Venus flytrap (VFT) domains.^57^ Through these mechanisms, TCSs can perceive and respond to a range of chemical and physical stimuli. In the absence of an activation signal, many HKs function as putative phosphatases of RRs, allowing a molecular reset of TCS signalling.^80–83^ Shifts in the equilibrium of kinase and phosphatase-active states are therefore necessary to balance the net physiological outcomes of TCS signalling.^84,85^ The molecular mechanisms of HK signal sensing, as it pertains to the structural dynamics of these mutually exclusive kinase and phosphatase competent states, have been extensively reviewed elsewhere.^8,58,86^ In brief, the activity of HKs is contingent on signal-dependent domain reorganisations, whereby the conserved kinase core adopts an asymmetrical homodimer configuration, containing both active and inactive catalytic subunits.^8^ The CA domain of the inactive conformation can bind to ATP, whilst the DHp domain remains accessible for phosphotransfer to the REC domain of an RR. In the active conformation, both the DHp and CA domains form a tight complex to catalyse in-*cis* or in-*trans* transfer of the γ-ATP phosphate onto the eponymous His site of the DHp domain, which is inaccessible to the RR.^8,86,87^ Bacteria also encode a range of non-TCS kinase signalling relay systems. A related group of non-TCS HKs are associated with the core signalling complexes of chemoreceptors in motile bacteria and archaea,^88^ which incorporate soluble cytoplasmic HKs (such as CheA in *E. coli*) as part of a supramolecular sensing array. Of note, several other HKs that are soluble enzymes, lacking transmembrane segments, have been identified.^34,89,90^

## Regulation of Ser/Thr protein kinases

Membrane-associated (and cytoplasmic) Hanks-type eSTKs serve regulatory functions in numerous cell processes and have also been implicated in the virulence of pathogens.^10,91–96^ eSTK-based signalling systems typically rely on the opposing activities of a protein kinase and an associated phosphatase, that (in contrast to HKs) can concurrently (de)phosphorylate Ser/Thr residues of a broad range of protein classes.^10^ However, mechanistic details of how branching eSTK signalling cascades are organised are relatively scarce. In addition to a surprisingly well-conserved Hanks-type fold in the kinase catalytic core,^45,94^ many eSTKs are buttressed with additional modular regulatory domains that serve as sensor regions, mediate protein–protein interactions and are sites of ligand binding. These include penicillin-binding and Ser/Thr kinase-associated repeats (PASTA) and forkhead-associated (FHA) domains that recognize phosphothreonines.^95,97,98^ In particular, extracellular sensory PASTA domains of transmembrane eSTKs (the “PASTA kinase” family), which can interact with ligands (usually peptidoglycan or peptidoglycan precursors) and induce activated kinase domain homodimers, are reasonably well characterised.^45,95,99^ PrkC, a membrane-associated eSTK of *B. subtilis*, contains three PASTA domains and plays crucial roles in spore germination, vegetative growth, modulation of cell wall synthesis and antibiotic resistance.^5,100^ PrkC activity is differentially regulated by bacterial growth phases. During the stationary phase, binding of peptidoglycan fragments to the extracellular PASTA domain results in PrkC dimerisation and activation of the cytosolic kinase domain (Fig. 2B),^101^ whilst during exponential growth, activation is attained by binding to the cell division proteins GpsB, DivIVA, and EzrA.^102^ Peptidoglycan binding by PASTA repeats of *S. aureus* STK similarly induces dimerization and activation of the kinase,^103^ which goes on to phosphorylate and inhibit several cell wall synthesis enzymes, such as FemX.^104^ PASTA domains also enable ligand-induced modulation of kinase activity and proper localisation of PknB from *M. tuberculosis*^105,106^ with dimerisation of the PknB kinase domain N-lobe resulting in allosteric activation of catalytic function.^107^

A common feature of eukaryotic protein kinases and several eSTKs is a requirement for phosphorylation of conserved sites within their putative activation loop to switch on catalysis. For example, the PASTA-kinase of *Enterococcus faecalis*, IreK, autophosphorylates at three positions on the activation loop (Thr^163, 166, and 168^) and an additional site on the C-lobe in response to cephalosporin treatment, resulting in enhanced activity.^108^ Autophosphorylation of activation loop amino acid residues appears to be a conserved regulatory mechanism for many eSTKs, including PrkA of *Listeria monocytogenes*,^109^ PrkC from *B. subtilis*,^110^ YegI of *E. coli*^111^ and PknB from *M. tuberculosis*.^112^ The molecular basis of mycobacterial cytoplasmic PknG activation is also predicted to involve N-terminal domain autophosphorylation, which creates a recruitment site for the substrate GarA, and displaces a substrate-occlusive N-terminal rubredoxin domain.^113^ Phosphorylation of juxtamembrane regions of PknG is also predicted to initiate interaction with the cell wall recruitment protein, FhaA.^114^ Other unique modes of eSTK regulation have also been discovered, including for YabT of *B. subtilis*, which is essential for a robust DNA-damage response, and harbours a DNA-binding motif that activates the kinase upon ds- or ss-DNA binding.^115^

## Antagonistic regulation of kinase activity by phosphatases

A responsive signalling network requires rapid and reversible addition of phosphate moieties to target proteins to selectively ‘switch on’ and ‘switch off’ signalling cascades. Across life, this involves the concerted opposing actions of protein kinases and protein phosphatases. For this purpose, dual-activity HKs can adopt phosphatase-competent states to ‘turn-over’ their signalling outputs. Moreover, RR dephosphorylation can be further accelerated by the actions of dedicated phosphatases, including CheZ of *E. coli*, Rap and Spo0E of *B. subtills*, and CheX of *Borrelia burgdorferi*.^58^ Bacteria have also evolved additional mechanisms to degrade phosphate-dependent signals in response to environmental cues. For example, bacterial phytochrome photoreceptors usually transmit photosensory input through a HK TCS system,^82^ including Agp1 bacteriophytochrome from *Agrobacterium fabrum* which exhibits light-sensitive HK-autophosphorylation activity.^116^ In contrast, no kinase activity has been detected for a structurally homologous bacteriophytochrome, DrBphP, from *Deinococcus radiodurans* which functions exclusively as a light-dependent phosphatase for the RR DrRR.^117^ In fact, bespoke bacterial phosphatases are prevalent in bacterial genomes, particularly in the context of eSTK- Ser/Thr phosphatase (STP) pairs^10,14,93,118^ and even arginine phosphorylation is counteracted by a cognate phosphatase, YwlE.^119^*M. tuberculosis* expresses 11 eSTKs (PknA-L, two of which, PknG and PknK, are soluble proteins) which collectively phosphorylate a wide range of protein substrates and play a dominant role in bacterial signalling, virulence and viability.^7,120–122^ Full activity of the membrane-anchored eSTK, PknB, depends on phosphorylation of two activation loop residues (Thr^171^ and Thr^173^), which can be reversed by dephosphorylation by the cognate STP, PstP.^112^ PstP is the only *M. tuberculosis* phosphatase discovered to-date and is predicted to dephosphorylate all members of the PknA-L family and their substrates.^45^ Interestingly, phosphorylation of PstP by PknA/B also regulates its catalytic activity, suggesting a regulatory feedback loop.^123,124^

The conserved *S. aureus* eSTK-phosphatase pair (STK-STP) is co-transcribed on a regulatory operon, with STP dephosphorylating STK.^125,126^ Notably, STK-STP has been implicated in controlling expression of secreted virulence factors, including hemolysins α, β and γ, which are drivers of *S. aureus* infection^127^ and the kinase/phosphatase pair also have antithetical activities on cell wall biosynthesis.^104^*Streptococcus agalactiae* phosphatase, Stp1, is also required for appropriate regulation of Stk1 eSTK function.^128^ eSTK-STP pairs of *Streptococci*, including Stk1-Stp1 in *S. agalactiae*, Stk-Stp in *S. pyogenes*, StkP-PhpP in *S. pneumoniae*, and PknB-PppL in *S. mutans* are similarly co-transcribed, to ensure integrative reversible control of Ser/Thr phosphate-signalling.^118^ An antagonistic relationship has also been observed between an eSTK of *Pseudomonas aeruginosa*, PpkA (which is essential for assembly of a type-VI secretion system [H-T6SS] and secretion of hemolysin-coregulated protein 1 [Hcp1]) and PppA, a Ser/Thr phosphatase.^129^ The lifecycle of *Bacillus anthracis*, the aetiological agent of human anthrax, comprises vegetative and sporulating phases. Spore germination is partly controlled by the co-expressed kinase-phosphatase pair PrkC-PrpC,^101^ with PrkC kinase activity inhibited by PrpC-dependent dephosphorylation. Curiously, PrpC is a dual specificity phosphatase capable of removing a phosphate moiety from Ser, Thr and Tyr residues of PrkD and PrkG kinases.^130^ Likewise, although a putative tyrosine kinase of *P. aeruginosa* has yet to be discovered, TpbA is also believed to be a novel dual specificity Try-phosphatase that negatively regulates biofilm formation in *P. aeruginosa*.^131^

## Regulation of kinase function through protein binding and subcellular distribution

Interactions with accessory proteins can determine the catalytic outputs of bacterial kinases, either through direct modulation of catalysis or organisation of their spatial distribution. For example, the McsAB^*B. subtilis*^ arginine kinase holoenzyme complex is comprised of the McsB kinase domain and an allosteric activator subunit, McsA.^132^ As previously discussed, interaction of the eSTK PrkC^*B. subtilis*^ with GpsB, DivIVA, and EzrA is responsible for appropriate localisation and activation of the kinase. Interestingly, PrkC is variably regulated by GpsB, which can function as both an activator and suppressor of PrkC, with the phosphorylated form of GpsB (itself a substrate of PrkC) providing a negative feedback loop to PrkC activity.^102^ EmbR is a transcriptional regulator, and a substrate protein of the mycobacterial eSTK, PknH. Curiously, a structural homolog, EmbR2 (which is a substrate of PknE and PknF) physically interacts with and inhibits PknH.^133^ Accessory proteins, GlnX and GlnH, are also required for PknG activation (a cytoplasmic eSTK of mycobacteria that lacks extracellular or transmembrane sensing regions) in response to extracellular amino acid concentration.^134^ HipA adopts an atypical protein kinase fold,^135–137^ and has been identified in the genomes of *E. coli*, and several paralogs are also found in other Gram-negative bacteria.^138^ HipA functions as a dormancy-driving toxin by inhibiting protein synthesis and is allosterically suppressed under normal growth conditions *via* direct complexation with the HipB antitoxin.^136,139^ Remarkably, HipA activity is also negatively regulated by intermolecular autophosphorylation of Ser^150^, which stabilises a P-loop ‘out-state’ and disrupts the ATP-binding pocket.^140^ HipT of *Legionella pneumophila*, which has homology to the N-terminal region of HipA, similarly adopts an inactivate conformation when in complex with its cognate antitoxin, HipS, which leads to the steric occlusion of ATP from the kinase core.^138^

HKs can similarly be regulated by accessory proteins. For example, two *B. subtilis* HKs, KinA and KinB, are essential for initiation of sporulation, and are respectively inhibited by Sda and KipI, which are predicted to repress autokinase activity by sterically blockading interaction between the CA and DHp subdomains.^141,142^ In contrast, binding of the accessory protein, PtsN to the DHp domain of the KdpD HK of *E. coli* stimulates *trans*-autophosphorylation between protomers of a KdpD dimer.^143^ Interaction with protein subunits can also shift the equilibrium of TCS kinase and phosphatase states. For example, the core SaeRS TCS of *S. aureus* is composed of a sensor kinase (SaeS), RR (SaeR), and two auxiliary proteins, SaeP and SaeQ, which regulate genes associated with biofilm formation.^144^ SaeQ and SaeP form a complex with SaeS and induce a phosphatase-competent state in the HK, effectively silencing signalling by the TCS. Finally, HK sensing of environmental stimuli (and subsequent activation) can also be mediated through accessory proteins. For example, the BceS HK of *B. subtilis* forms an activating intermolecular sensory complex with BceAB.^145^ In this system, the BceS sensor kinase is indirectly activated by the peptide-based antibiotic, bacitracin, through a flux-sensing mechanism which detects conformational cycling of the BceAB transporter.^146,147^ The PhoQP system can also be indirectly regulated by the acid sensing EvgSA system of *E. coli via* an intermediary membrane protein, SafA. SafA expression is induced following activation of EvgSA, and directly interacts with the PhoQ periplasmic sensor domain to stimulate autophosphorylation.^148–150^ The Rcs envelope stress response phosphorelay system (comprising RcsC HK, RcsD phosphotransfer protein, and RcsB RR) is conserved in enterobacteria (reviewed in ref. 66, 67 and 151) and is regulated by the outer-membrane sensor lipoprotein, RcsF.^152^ Under normal growth conditions, another inner membrane protein, IgaA, represses the RcsCDB phosphorelay cascade through an inhibitory interaction with RcsD. However, detection of envelope stress by RcsF induces complex formation with AgaA, relieving repression and resulting in autophosphorylation of RcsC.^153,154^

Subcellular localisation can also be critical for kinase function. Cell pole organising protein PopZ of *Caulobacter crescentus* self-assembles into a polymeric superstructure that recruits several signalling protein clients (such as the cell fate HKs CckA and DivJ) into a regulatory hub that coordinates cellular polarity.^155,156^ DivJ expression at the cell poles is critical for propagating chromosomal replication and stalk appendage biogenesis,^157^ and this is controlled by PopZ-dependent recruitment factor, SpmX.^156^ A second *C. crescentus* scaffold protein, PodJ, assembles at the opposite cell pole to PopZ and sequesters several additional proteins, including the HK PleC *via* its sensory PAS domain.^158^ CckA and PleC of *C. crescentus* are bifunctional HKs that oscillate between kinase and phosphatase activity.^159^ CckA exhibits density-dependent activity coordinated through its tandem PAS domains;^159^ functioning as an indirect kinase of the CtrA transcription factor when accumulated at high density at a newly formed cell pole,^160^ but transitioning to a phosphatase of CtrA in the presence of high levels of the secondary messenger c-di-GMP (cdG).^160–162^ Recruitment to the PopZ microdomain, which defines CckA surface density, is partly regulated through complexation with a catalytically inactive pseudohistidine kinase, DivL.^163–165^ Curiously, DivL is capable of transforming information relating to subcellular localisation into conformational switches that can toggle CckA between its kinase or phosphatase modes, suggesting a highly dynamic regulatory profile for this HK complex.^166–168^ PleC is also mechanistically density-regulated through PAS domains and is recruited to polar PodJ biomolecular condensates, which simultaneously stimulates PleC phosphatase function and suppresses kinase activity.^158^ Spatial organisation may also be an evolutionary strategy to limit cross-talk between TCSs, and has been observed in *Rhodobacter sphaeroides* and for the CheZYA chemotaxis system of *E. coli*, as reviewed by Sourjik and Armitage, 2010.^169^

## Cross-talk between bacterial phosphorelay systems

**Fig. 3:**
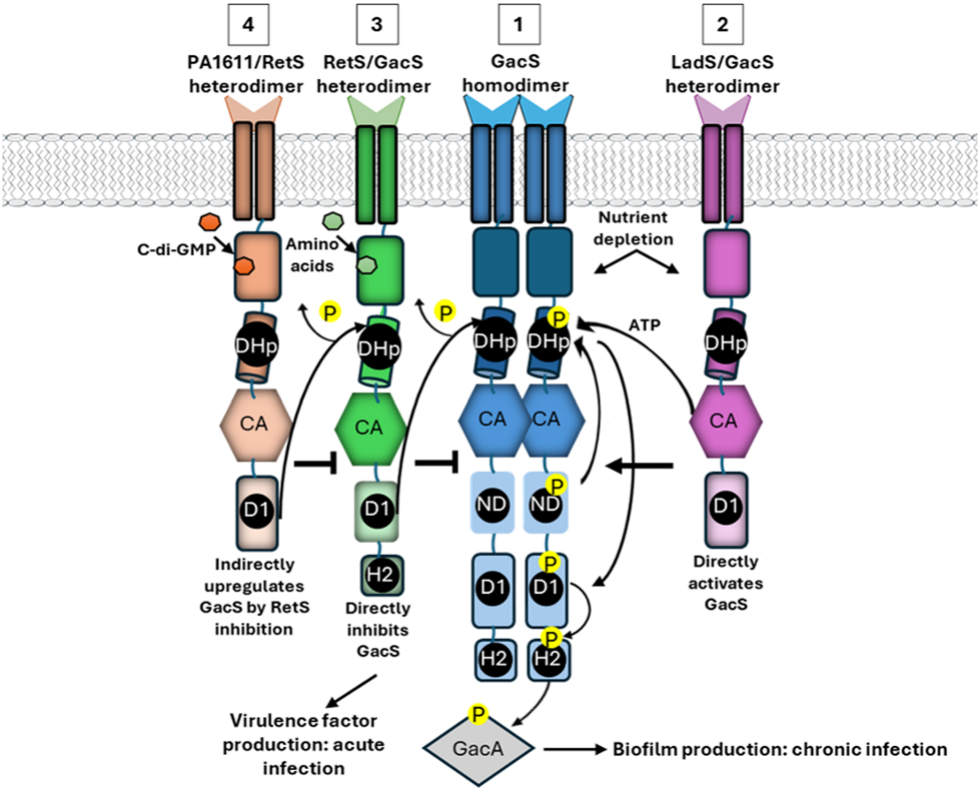
Cross-talk between GacS/A and other TCSs in *P. aeruginosa*. (1) Periplasmic or cytosolic sensor domains respond to environmental or cellular signals that cause GacS homodimerization. The ND and CA domains facilitate autophosphorylation of a conserved His in the DHp domain. GacS contains an additional receiver (D1) domain, which becomes phosphorylated by intramolecular phosphotransfer. The phosphate is subsequently shuttled to the H2 domain and finally GacA, the RR. (2) LadS positively regulates GacS function, enhancing kinase activity and supporting chronic infection characteristics. (3) RetS forms a complex with GacS, inhibiting its kinase activity thereby suppressing GacA phosphorylation, leading to enhanced expression of acute virulence factors. (4) PA1611 binds to RetS, preventing its inhibitory interaction with GacS. By sequestering RetS, PA1611 allows GacS to maintain its kinase activity, facilitating GacA phosphorylation and promoting chronic infection pathways.

Cognate HK-RR pairs are usually highly monogamous and are generally co-expressed under a single promotor.^170,171^ As such, encoded TCS paralogs typically serve distinct cellular functions through linear signal transmission processes.^61,171^ Retention of signal fidelity is primarily dictated by specificity-determining amino acids that flank the conserved phosphotransfer/acceptor sites in the DHp and REC domains.^172^ However, convergence between non-cognate TCS pairs has also been observed in some bacteria.^173–176^ For example, in *E. coli*, NarQ can target its cognate RR, NarP, and also cross-phosphorylate NarL of the NarX-NarL TCS axis,^177^ and CreC can interact with PhoB, the cognate RR of PhoR.^178^ Far from representing an evolutionary redundancy, crosstalk between TCS may enhance the complexity of responses to individual input events, and promote bacterial growth, metabolism and survival.^173,176^ Relaxed specificity is potentially less detrimental in unorthodox ‘hybrid’ TCS, where spatial tethering of transmitter and receiver domains effectively increases their local concentrations to one another.^179–181^ For example, the GacSA hybrid TCS of *P. aeruginosa* is part of a multi-kinase communication network with LadS, RetS and PA1611 hybrid HKs^182,183^ (Fig. 3). Following signal-mediated activation, GacS autophosphorylates at His294 (H1), and the phosphate moiety is then shuttled to Asp717 (D1) and His863 (H2).^18^ Heterodimersiation of RetS and GacS downregulates GacSA signal transduction by inhibiting GacS autophosphorylation, siphoning phosphate groups from the DHp site, and through direct dephosphorylation of the GacS D1 site by RetS.^184,185^ Moreover, RetS-dependent inhibition can subsequently be relieved through formation of a competitive RetS-PA1611 heterodimer.^186^ In contrast, LadS forms a multi-component phosphorelay system with GacS, with the transmitter and REC domains of LadS facilitating *trans*-phosphorylation of the GacS H2 domain.^182^ Through these mechanisms, RetS and LadS can reciprocally regulate virulence factor expression controlled by the GacSA regulon and influence initiation of chronic *P. aeruginosa* infection.^182^ Cross-communication has also been detected between HssSR and HitSR of *B. anthracis*,^187^ NtrBC and NtrYX of Rhodobacter capsulatus,^188^ RscS and SypF of *Vibrio fisheri*^189^ HpArsRS and HpNikR of *Helicobacter pylori*^190^ and GraSR and ArlSR of *S. aureus*.^191^ Interestingly, RR heterodimers have also been described that can enable differential coactivation of target genes, as reviewed by Agrawal *et al.*, 2016.^172^

HKs and the other major phosphorelay system, eSTKs, may also intertwine to potentiate the output of adaptive signalling responses (Fig. 2B). This is perhaps not surprising given recent studies revealing that eSTKs exert a broad regulatory influence; phosphorylating 80% of the *M. tuberculosis* proteome and modulating expression of ∼30% of its genes.^7^ Such an expansive regulatory footprint may facilitate overlapping regulons between eSTKs and TCS. Indeed, 170 *O*-phosphorylation sites were identified on TCSs of *M. tuberculosis*, suggesting extensive intersection of these two phosphosignalling pathways.^192^ Notably, phosphorylation of the *M. tuberculosis* TCS-HK, NarS, at Thr^380^ by an eSTK, PknL, was sufficient to stimulate its autokinase activity and downstream signalling.^192^ The AlgRZ TCS, which is comprised of AlgR, and it's putative HK AlgZ, is an important regulator of *P. aeruginosa* gene expression and pathogenesis.^193^ Strikingly, Ser^143^ of the RR, AlgR, is also phosphorylated by the eSTK Stk1, which adjusts its physiological functions.^194^ Cross talk between STK of *S. aureus* and TCSs involved in cell wall metabolism has also been described, through direct phosphorylation of several Thr residues in VraTSR,^195^ WalRK,^196^ and GraSR.^197^ The StkP-PhdP eSTK-STP pair of *S. pneumoniae* can also reversibly regulate the RRs, RitR,^198^ RR06,^199^ and PknB^*M. tuberculosis*^ can downregulate DevSR and SenX3/RegX3 signalling by phosphorylating their RRs.^200,201^ Fascinatingly, another *P. aeruginosa*, RR PA3346, contains an N-terminal receiver domain and an active STP domain that dephosphorylates Ser^56^ of another RR, PA3347. The Ser/Thr phosphatase activity of PA3346 is in turn potently stimulated following phosphorylation of the RES domain by a HK, PA2824.^202^ Direct modulation of HK kinase activity by eSTKs has also been described. For example, the CroS HK of the opportunistic pathogen, *Enterococcus faecalis*, is phosphorylated at a Thr by a transmembrane PASTA-eSTK, IreK, thereby enhancing HK activity. Of note, this site (Thr^346^) is situated in the ATP lid of the CroS CA domain, a crucial structure for appropriate nucleotide positioning and phosphate exchange during autophosphorylation.^203^ An eSTK of *B. subtilis*, YbdM, was also reported to directly phosphorylate the sensing domain of an atypical cytoplasmic HK, DegS, and enhance its kinase activity *in vitro*.^204^ Fascinatingly, the *S. aureus* tyrosine protein kinase CapAB (which positively regulates capsule biosynthesis) can be negatively regulated by PknB-dependent phosphorylation^205^ suggesting communication between eSTK and tyrosine kinase phosphotransfer networks.

## Regulation of kinases by cofactors, secondary messengers, and quorum sensing

Much like their eukaryotic counterparts, bacterial kinases are also regulated by cofactors that can induce conformational changes and interdomain interactions or augment catalytic function. Coordination of haem or flavin adenine-dinucleotide (FAD) cofactors by HK PAS domains enables sensing of cellular redox state. For example, the kinase activity of FixL (found in nitrogen-fixing rhizobia), is potently inhibited by O_2_ binding to a PAS-B located haem group.^206^ Redox sensing cysteines have also been discovered in the CA domain of the *S. aureus* HK, SrrB, which form a unique intramolecular disulfide bond that tunes autokinase function.^207^ Furthermore, oxidation or *S*-nitrosylation of a *Salmonella enterica* RR, SsrB, at Cys^203^ lowers DNA-binding affinity and thus signalling by the SsrA/SsrB TCS,^208^ which may represent a defensive adaptation to promote *Salmonella* fitness when exposed to nitric oxides (NOs) produced by the host immune response. Similarly, Cys^67^, located in the REC domain of the redox-sensitive WalR RR, can be *S*-nitrosylated to modulate WalKR signalling and promote *S. aureus* NO-mediated vancomycin resistance.^209^ The cyanobacterium *Synechocystis* sp. PCC 6803, encodes six eSTKs (SpkA-D, G), one pseudokinase (SpkE), and five atypical kinases (SpkH-L). Of note, SpkB was shown to be inactivated by oxidation of an N-terminal Cys motif, as an adaptation to oxidative stress tolerance.^210^ As previously discussed, the secondary messenger c-di-GMP binds within the interface between the PAS-B site and CA domain of the HK, CckA, to stimulate phosphatase activity.^159,211^ In contrast, the kinase activities of ShKa, a cytosolic hybrid HK of *C. crescentus*, and RavS, a HK of *Xanthomonas campestris*, are increased following binding to c-di-GMP.^212,213^ The activity of a DNA-damage responsive eSTK of the extremophilic bacteria *Deinococcus radiodurans*, RqkA, is also enhanced by binding to pyrroloquinoline-quinone (PQQ).^214^

*S. aureus* biofilm formation is partially regulated by intercellular communication and density sensing, commonly known as quorum sensing.^215^ In this manner, bacteria can detect changes in population numbers based on the local concentration of specific autoinducer signals. Direct binding of an autoinducer peptide (AIP) to the AgrC HK stimulates phosphorylation of the RR AgrA, resulting in positive regulation of toxin genes, autoinduction of AIP biosynthesis, and reduced expression of several surface adhesins, as reviewed by Jenul & Horswill, 2019.^216^ The Lux system in *Vibrio* species is another well-studied TCS that is regulated by quorum sensing.^217^ Low autoinducer concentrations at low cell density induces LuxQ activity and results in phosphorylation of the LuxO RR. In contrast, high autoinducer concentrations (detected by the periplasmic receptor LuxP) activate HK phosphatase activities and reverts the TCS to a pre-activated state.^218^

## Regulation of secreted effector kinases

In order to establish an infection and subvert host defences, pathogenic bacteria can also deploy an arsenal of signal pathway-hijacking virulence effectors, including eSTKs, into the cytoplasm of target cells.^219^ PknB^*M. tuberculosis*^ (and the phosphatase SapM) was the first discovered effector eSTK, which functions to promote intracellular survival of the bacteria in macrophages by blocking phagosome maturation.^220,221^ Since then, secreted bacterial effector kinases have been identified in the genomes of numerous pathogens, including *Yersinia*, *Salmonella*, *Legionella* and *Shigella*^219^ but little is understood about how they are regulated. To avoid premature activation of cytotoxicity, the activities of virulence factors and exotoxins are extremely tightly controlled, only ‘switched on’ following secretion or exposure to specific host-derived activation signals. Similar host-dependent activation mechanisms have also been observed for a handful of effector eSTKs. *Legionella pneumophila* (L.p.) is an opportunistic intracellular pathogen of human alveolar macrophages and the causative agent of Legionnaire's disease. L.p. translocates over 300 survival-promoting effector proteins, including at least 6 experimentally validated eSTKs (LegK1-4, LegK7, and Lem28/Lpg2603) into invaded host cells *via* the Dot/Icm type IV secretion system (T4SS).^222–231^ Fascinatingly, LegK7, which manipulates the host Hippo pathway by functioning as a molecular mimic of Mammalian Ste20-like kinases 1/2 (MST1/2), is allosterically activated by N-terminal binding to a host scaffold protein, MOB kinase activator 1A (MOB1A).^227,228^ Similarly, Lem28 is a remote member of the protein kinase superfamily that is allosterically activated by the eukaryotic-specific ligand inositol hexaphosphate (IP_6_).^230^ Members of the pathogenic *Yersinia* genus secrete an eSTK domain containing protein, YpkA, which directly phosphorylates the heterotrimeric Gαq protein and vasodilator-stimulated phosphoprotein (VASP) to disrupt macrophage cytoskeletal dynamics and phagocytosis.^232,233^ YpkA is delivered into host cells in an inactive form *via* a type III secretion system, where it is allosterically activated following actin binding to the actin binding domain (ABD) and undergoes extensive autophosphorylation.^234,235^ The *Shigella* type-III secretion system effector OspG is an atypical serine/threonine protein kinase that lacks several canonical structural features, including a regulatory activation loop.^236^ Despite the extensive degradation of the catalytic core, OspG exhibits intrinsic kinase activity, but only upon binding to components of the host ubiquitin system.^236^ In contrast, homologous atypical kinases of *E. coli*, NleH1, and NleH2, display ubiquitin-independent autophosphorylation which promotes interaction with target proteins.^236^

## TCSs as potential therapeutic targets for the treatment of microbial infections

Multi-drug resistant (MDR) bacterial infections rank globally amongst the leading causes of mortality, and are projected to cause 40 million deaths by 2050.^237^ Most clinically approved antibiotics are derivatives of small molecules that disrupt a narrow range of essential microbial biosynthetic pathways. Compounds exhibiting alternative modes of action are scarce,^238^ which has exacerbated the spread of MDR bacteria. Accordingly, the many crucial roles that TCSs and kinases serve in bacterial growth, viability and pathogenic processes (including antibiotic resistance mechanisms and biofilm formation) make them attractive alternative therapeutic targets to develop novel antimicrobials. Moreover, the CA and DHp domains of TCS (which are crucially absent in mammalian signalling pathways) are highly conserved across the bacterial kingdom,^84,239^ potentially enabling development of compounds with broad-spectrum activity.^240,241^ In this final section we discuss some recent advances in kinase-targeting antimicrobial drug strategies.

Several studies have investigated and confirmed TCS inhibition by small-molecule compounds. For example, WalK is an essential HK conserved among Gram-positive bacteria, including *S. aureus* and *B. subtilis*.^242^ Walkmycins A, B, and C were demonstrated to exhibit strong antibacterial activity against *B. subtilis* (a model organism for studying pathogenic *B. anthracis* and *B. cereus*) and walkmycin B was revealed to specifically inhibit WalK autophosphorylation.^243^ Interestingly, walkmycin C was also an effective inhibitor of three other *Streptococcus mutans* HKs: VicK, CiaH, and LiaS *in vitro*,^244^ and isatin derivatives also exhibit potent antimicrobial activity against *S. aureus*, which was attributed to direct binding to and inhibition of WalK.^245^ Other inhibitors of HK autophosphorylation have now been identified for several different bacterium, targeting VicK and AgrC of *S. pneumoniae*,^246^ DosS and DosT of *M. tuberculosis*,^247^ and PhoQ of *S. typhimurium*,^248^ suggesting that TCSs are viable antibacterial therapeutic targets.

Most identified TCS-targeting inhibitors, including walkmycin C,^244^ are functional ATP-competitive molecules that ‘blockade’ ATP binding and thus HK catalysis (HKs such as HK853^*Thermotoga maritima*^, CheA^*E. coli*^ and VicK^*S. pneumoniae*^ can all be inactivated by ATP obstruction^249^). The ATP-binding pocket of the HK CA domain adopts a distinctive α/β sandwich, consisting of four conserved regions (the N-box, the G1-box, the G2-box, and the G3-box) and a highly variable ATP-lid.^250,251^ This structural architecture is known as the Bergerat fold, and is also observed in the ATP-binding domains of the diverse GHKL protein superfamily, which also includes DNA gyrases, HSP90, and the MutL mismatch repair enzyme.^250^ Guarnieri *et al.* (2008) demonstrated that the human HSP90 inhibitor, radicicol, binds to the ATP-binding pocket of the *Salmonella* HK, PhoQ (albeit weakly).^252^ Since then, optimised derivatives of ATP-competitive HSP90 inhibitors have been explored as potential HK active agents.^251,253^ For example, a fragment of an established HSP90, 3,4-diphenylpyrazole (DPP)-based inhibitor was shown to bind to the CA domain of CheA from *Thermotoga maritima*, and several DDP analogues are effective inhibitors of EnvZ^*E. coli*^, PhoQ^*Salmonella typhimurium*^, and CckA^*C. crescentus*^.^251,253^ Repurposing human protein kinase inhibitors (for which there are ∼100 FDA approved clinical compounds) is another desirable strategy to identify novel therapeutic agents to target bacterial kinases. Carabajal *et al.* (2020) screened 686 compounds from the Published Kinase Inhibitor Set (PKIS [by GlaxoSmithKline]), and identified quinazoline-based ATP-competitive inhibitors of *S. typhimurium* PhoQ that repressed autokinase kinase activity.^248^ However, despite these promising results, potential toxicity associated with ‘off-target’ inhibition of human kinase signalling pathways may necessitate a more refined, structure-guided design approach to develop selective inhibitors which only target bacterial HKs before such agents can be considered therapeutically viable.

Interestingly, several non-ATP competitive bacterial kinase inhibitors have also been discovered. The HK PhoQ (of the conserved PhoP/PhoQ TCS) plays pivotal roles in *Salmonella*, *Shigella*, and *Pseudomonas* pathogenesis, regulating virulence factor production and bacterial survival within host cells.^254^ Moreover, PhoP/PhoQ contributes to ‘last-resort’ polymyxin antibiotic resistance by indirectly activating the PmrA/PmrB TCS.^255,256^ Fascinatingly, PhoQ (*Salmonella typhimurium*) HK activity can be repressed by allosteric targeting its second transmembrane region using a hydrazone derivative compound, *N*′-(thiophen-2-ylmethylene)benzohydrazide (designated A_16_B_1_).^257–259^ Watanabe *et al.*, (2012) also identified signermycin B as a potent allosteric inhibitor of WalK that disrupted autokinase activity by sterically blocking the HK dimerization domain.^260^ Moreover waldiomycin (and a methyl ester derivative) can effectively inhibit WalK from both *S. aureus* and *B. subtilis* (and PhoR and ResE from *B. subtilis*) by binding to the phosphoacceptor region and blocking autophosphorylation.^261,262^

## Conclusions

TCSs and bacterial kinases represent a promising frontier for the development of novel therapeutics for microbial infections, offering a versatile platform for addressing both antimicrobial resistance and bacterial virulence. Phosphate-based PTMs, regulated by kinases and phosphatases, are essential for bacterial homeostasis, viability and virulence and clinical compounds with the ability to inhibit conserved domains across multiple protein kinases could provide broad-spectrum antimicrobial activity. Conceptually, TCS inhibitors also show promise as therapeutic adjuvants, potentially synergizing with conventional antibiotics to overcome resistance mechanisms.^263^ For example, compounds like PhoQ-targeting A_16_B_1_ can enhance the bactericidal activity of polymyxins.^259^ However, despite significant interest in this area, no bacterial kinase-targeting small molecule have advanced to clinical approval. To progress this area of research, a combination of structure-guided drug design and innovative screening methodologies will be essential to develop optimised and selective kinase-targeting compounds with robust antimicrobial activity.

## Author contributions

Writing – original draft: DPB, DMF, DMC & CW; writing – review and editing: DPB, DMF, DMC & CW; visualisation: DMF, DMC & DPB.

## Conflicts of interest

There are no conflicts of interest.

## Data Availability

No primary research results, software or code have been included and no new data were generated or analysed as part of this review.
